# Association between the triglyceride-glucose index and calcified aortic stenosis in elderly patients: a cross-sectional study

**DOI:** 10.1038/s41598-023-42206-x

**Published:** 2023-09-11

**Authors:** Zhihui Hu, Tiantian Xiong, Chunling Chen, Tao Chen, Ming Li, Jia Liang, Kunying Chen, Jialing Zhang, Xu Chen, Qi Chen, Guoming Li

**Affiliations:** 1 The Affiliated Hospital of Guangdong Medical University, Zhanjiang, China; 2 The Third People’s Hospital of Huizhou, Huizhou, China; 3 Guangdong Medical University, Zhanjiang, China

**Keywords:** Cardiology, Cardiovascular biology, Calcification

## Abstract

Insulin resistance (IR) is associated with a variety of cardiovascular diseases, but there are few studies on the correlation between IR and calcified aortic stenosis (CAS). In this study, the triglyceride-glucose (TyG) index, which reflects IR, was used to investigate the correlation between IR and CAS. The study included 183 elderly patients who were diagnosed with CAS by transthoracic echocardiography. The patients were matched 1:1 according to age and sex, and elderly patients who were hospitalized during the same period and underwent transthoracic echocardiography without aortic stenosis were included as the control group. The relationship between the TyG index and CAS was analyzed by a multivariable logistic regression model, curve fitting and trend test. Multivariate logistic regression analysis showed that the TyG index as a continuous variable was negatively associated with CAS (P < 0.001); trend tests and curve fitting further supported this association. Our study showed that the TyG index was negatively associated with CAS in elderly patients, which may be related to the impairment of insulin receptors and signaling pathways in IR.

## Introduction

Aortic stenosis is the most common valvular disease of the heart. It is usually caused by congenital bicuspid aortic valves and rheumatic and degenerative trilobal calcification in old age. The prevalence of aortic stenosis is increasing due to population aging. Calcified aortic stenosis (CAS) is a common cause of aortic stenosis in elderly individuals, and its main pathological manifestations are calcification and fibrosis of the valve tissue, resulting in leaflet rigidity, stenosis of the flap opening, increased left ventricular afterload, ventricular remodeling, fibrosis and diastolic dysfunction in the left ventricle, and eventually a series of clinical symptoms, such as angina pectoris and dyspnea^1^. It is currently believed that CAS is divided into two stages of progression. The first stage is similar to the occurrence of atherosclerosis. The second stage is the reproduction and calcification stage, which is mainly dominated by valvular interstitial cells (VICs) in the aortic valve layer, which are activated by oxidized lipoproteins and inflammatory cytokines, differentiated into myoblasts and osteoblasts^2,3^, and subjected to osteoblastic changes.

Insulin resistance (IR) refers to the deficiency of insulin-mediated glucose metabolism in tissues such as muscle, fat and liver and is one of the early manifestations of a series of human diseases, including type-2 diabetes mellitus and cardiovascular disease^4^. The hyperinsulinemic-euglycemic clamp (HIEC) is the gold standard method to evaluate IR. In addition, the homeostatic model assessment for IR (HOMA-IR) is often used to assess IR, but the application scenarios of these two methods are limited by the fact that fasting insulin levels are rarely detected in daily clinical practice or large-scale epidemiological investigations. The triglyceride-glucose (TyG) index is a simple and inexpensive indicator to estimate IR, which has been shown to have comparable accuracy with HECT and HOMA-IR^5^ and is considered to be a reliable surrogate marker for IR. In cardiovascular disease-related studies, the Tyg index is positively correlated with cardiometabolic risk factors such as cholesterol, uric acid, and waist-to-hip ratio^6^ and is a common risk factor in cardiovascular diseases such as coronary artery disease, heart failure and peripheral artery disease, as well as osteoporosis and bone mineral density ^7–12^. In a prospective study of 141,243 participants from 22 countries, the TyG index was found to be significantly associated with future cardiovascular mortality, myocardial infarction, stroke, and type-2 diabetes, suggesting that IR plays a contributing role in the pathogenesis of cardiovascular and metabolic diseases^13^. However, there are few studies on the correlation between the Tyg index and CAS.

Therefore, the purpose of this study was to explore the correlation between the TyG index and CAS in the elderly population to provide different viewpoints on the occurrence and development of CAS.

## Methods

### Study population

This cross-sectional study included elderly patients (aged ≥ 60 years) admitted to the Department of Cardiovascular Medicine, Affiliated Hospital of Guangdong Medical University from August 2019 to August 2022 and diagnosed with CAS by transthoracic echocardiography. The patients were matched 1:1 according to age and sex. Elderly patients (aged ≥ 60 years) who were hospitalized during the same period and underwent transthoracic echocardiography without aortic stenosis were included as the control group. The patients with CAS were further divided into mild stenosis, moderate stenosis and severe stenosis according to the results of cardiac color ultrasound imaging. This study was approved by the Ethics Committee of the Affiliated Hospital of Guangdong Medical University and conducted in accordance with the ethical principles of the Declaration of Helsinki. The requirement for informed consent from the study subjects was waived by the IRB of the Affiliated Hospital of Guangdong Medical University due to the retrospective study design.

### Inclusion criteria

The diagnosis and classification criteria for CAS met the American College of Cardiology/American Heart Association standards^6^. Mild aortic stenosis was defined as a maximum transvalvular flow (AV Vmax) in the main artery of 2.0–2.9 m/s or a mean transvalvular differential pressure (AV MP) < 20 mmHg. Moderate aortic stenosis is defined as a Vmax of 3.0–3.9 m/s or a mean differential pressure of 20–39 mmHg. Severe aortic stenosis was defined as transvalvular flow (Vmax) ≥ 4 m/s or mean transvalvular differential pressure ≥ 40 mmHg.

### Exclusion criteria

The following patients were excluded from the study: (1) patients with rheumatic heart disease; (2) patients with bilobated aortic valve deformity; (3) patients with severe aortic insufficiency or severe mitral valve disease; (4) patients with a history of valve replacement surgery; (5) patients with infective endocarditis; (6) patients with malignant tumors, mental illness, thyroid and parathyroid diseases; (7) patients with severe infection, shock and other life-threatening conditions; (8) patients with severe renal insufficiency or liver insufficiency; (9) patients with incomplete clinical data or laboratory test results.

### Clinical information collection

We extracted patients' clinical information from the electronic medical record system of the Affiliated Hospital of Guangdong Medical University. The laboratory indicators were the results of the patient's examination within 24 h after admission. Patient clinical data were collected, including but not limited to sex, age, smoking history, BMI, hypertension, type-2 diabetes, atrial fibrillation, stroke, COPD, and hepatic and renal insufficiency. The levels of hemoglobin, red blood cells and platelets were measured by the European Sysmex XN3000 hematology analyzer. Glu, TG, TC, HDL-C, LDL-C, UA, urea, Cre, CK, CK Mb, LDH and Mb were determined by German Cobas 8000 biochemical analyzer spectrophotometry. Nt-proBNP and hypersensitive troponin were determined by a German Cobas e801. Cardiac ultrasound data measured by GE Vivid E95 USA: LVEF, transaortic maximum velocity, and mean pressure gradient between the LV and aorta. The data is collected by dedicated researchers.

### Data analysis

Empower (R) (www.empowerstats.com, X&Y Solutions, Inc., Boston, MA) and R (http://www.R-project.org) were applied to all statistical analyses in the study. Continuous variables are expressed as the mean (standard deviation) (normal distribution) or median (Q1–Q3) (skewed distribution). Categorical variables are described as the number or percentage. Continuity variables were analyzed using the T test or Kruskal‒Wallis rank sum test, and categorical variables were analyzed using the χ2 test or Fisher exact probability test. With the TyG index as the independent variable and CAS as the dependent variable, the correlation between the TyG index and CAS was analyzed by a univariate/multivariable logistic regression model and verified by a trend test. Curve fitting was used to examine the shape of the associations between the TyG index and CAS. Meanwhile, the control group was taken as the reference group, and multiple regression analysis was used to further analyze the relationship between different degrees of CAS and the TyG index. Statistical significance was set at α = 0.05.

### Ethics approval

This study was approved by the Ethics Committee of the Affiliated Hospital of Guangdong Medical University and conducted in accordance with the ethical principles of the Declaration of Helsinki.

### Consent to participate

The requirement for informed consent from the study subjects was waived by the IRB of the affiliated hospital of Guangdong Medical University due to the retrospective study design.

## Results

### Study population

**Figure 1:**
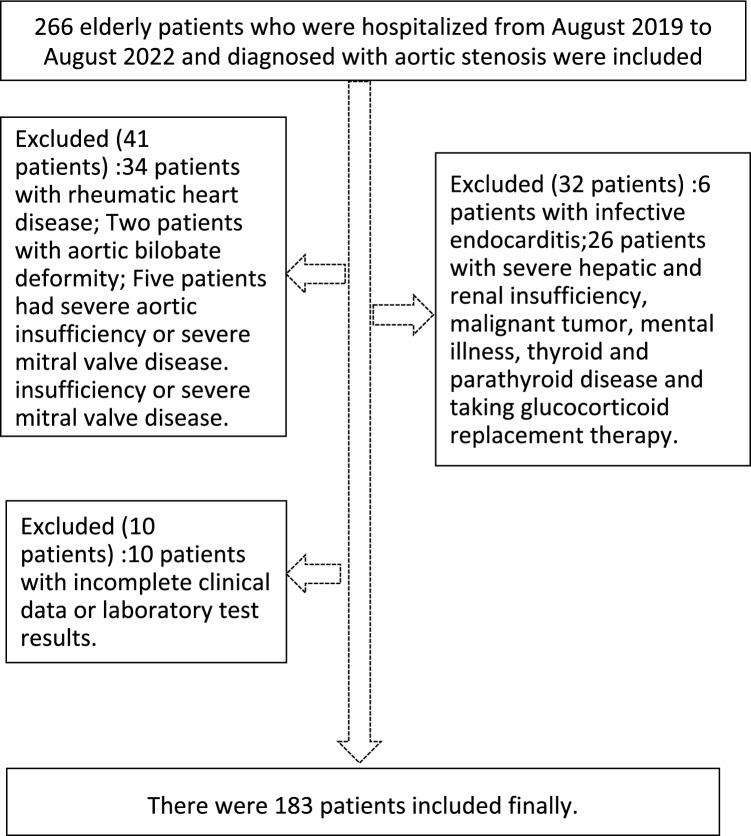
Flow chart of patient selection.

As shown in Fig. 1, 266 elderly patients admitted to the Department of Cardiovascular Medicine, Affiliated Hospital of Guangdong Medical University from August 2019 to August 2022 and diagnosed with major aortic stenosis by transthoracic echocardiography were included in the study. After excluding 34 patients with rheumatic heart disease; 2 patients with aortic bilobate deformity; 5 patients with severe aortic insufficiency or severe mitral valve disease; 6 patients with infective endocarditis; 26 patients with severe hepatic and renal insufficiency, malignant tumor, mental illness, thyroid or parathyroid disease or who were receiving glucocorticoid replacement therapy; and 10 patients with incomplete clinical data or laboratory test results, 183 patients were included. The total number of participants in the study was 366 (183 in the CAS group and 183 in the control group).

### Baseline characteristics of participants (N = 366)

As shown in Table 1, there were 183 patients in the CAS group (male: 104, female: 79). Moreover, smoking, drinking, T2DM, hypertension, hyperlipidemia, and chronic renal insufficiency stage 1–3 were not significantly different between the two groups (P > 0.05). The serum uric acid and NT-proBNP levels in the calcific aortic stenosis group were significantly higher than those in the control group (P < 0.05). Compared with the CAS group, systolic blood pressure, HbA1c, TyG index, LVEF, fasting blood glucose and triglycerides in the control group were higher (P < 0.05). The proportion of NYHA grade III/IV in the CAS group was higher than that in the control group (P < 0.001).

**Table 1:** Baseline data characteristics of the subjects (n = 366).

	CAS	Control	t/z/χ2	P-value
N(number)	183	183		
Age (years)	74.62 ± 7.50	74.62 ± 7.50	0.000	1.000
Heart rate (beats/min)	76.93 ± 16.32	76.69 ± 16.51	0.141	0.840
SBP (mmHg)	145.60 ± 23.20	138.31 ± 24.39	2.923	0.004
DBP (mmHg)	78.54 ± 13.45	71.49 ± 12.80	5.130	< 0.001
Weight (kg)	60.31 ± 11.29	56.95 ± 10.95	2.591	0.010
Height (cm)	161.33 ± 8.24	160.83 ± 7.47	0.206	0.837
HbA1c (%)	6.40 ± 1.45	6.06 ± 1.18	2.297	0.022
NT-proBNP (Ng/L)*	278.5 (82.9–1089.5)	1703.0 (455.6–6397.0)	-3.364	< 0.001
Hs-cTn (Ng/L)*	0.01 (0.01–0.02)	0.02 (0.02–0.04)	-1.693	0.092
CRP (mg/L)*	6.33 (2.91–12.40)	3.74 (2.38–8.07)	1.71	0.174
WBC (× 109/L)	7.36 ± 2.66	7.30 ± 2.41	0.191	0.994
creatinine (μmol/L)	89.13 ± 50.42	90.67 ± 29.83	-0.3558	0.722
SUA (μmol/L)	368.49 ± 117.43	405.81 ± 124.05	-2.940	0.003
FPG (mg/dl)	105.58 ± 37.32	95.45 ± 28.18	2.924	0.004
TC (mg/dl)	176.02 ± 49.62	171.64 ± 51.30	0.825	0.410
TG (mg/dl)	121.87 ± 79.77	107.42 ± 74.93	1.777	0.035
TygI	8.57 ± 0.62	8.37 ± 0.57	3.288	0.001
HDL-C (mg/dl)	47.44 ± 14.10	46.09 ± 12.60	0.960	0.338
LDL-C (mg/dl) *	2.77 (1.98–4.14)	2.78 (2.03–4.03)	-0.065	0.948
LVEF (%)	56.98 ± 9.02	52.85 ± 11.56	3.798	< 0.001
AV Vmax (m/sec)	–	4.08 ± 1.25		
AV MP (mmHg)	–	41.38 ± 23.13		
Gender (%)			0.000	1.000
Female	79 (43.17%)	79 (43.17%)		
Male	104 (56.83%)	104 (56.83%)		
Smoking (%)			0.000	1.000
NO	159 (86.89%)	159 (86.89%)		
YES	24 (13.11%)	24 (13.11%)		
Drinking (%)			0.555	0.456
NO	176 (96.17%)	173 (94.54%)		
YES	7 (3.83%)	10 (5.46%)		
Hypertension (%)			3.553	0.059
NO	77 (42.08%)	95 (51.91%)		
YES	106 (57.92%)	88 (48.09%)		
T2DM (%)			2.346	0.126
NO	138 (75.41%)	150 (81.97%)		
YES	45 (24.59%)	33 (18.03%)		
Coronary heart disease			10.503	0.001
NO	76 (41.53%)	107 (58.47%)		
YES	107 (58.47%)	76 (41.53%)		
Hyperlipidemia			0.659	0.417
NO	159 (86.89%)	164 (89.62%)		
YES	24 (13.11%)	19 (10.38%)		
Renal insufficiency (%)			0.261	0.609
NO	176 (96.17%)	174 (95.08%)		
YES	7 (3.83%)	9 (4.92%)		
NYHA Class (%)			38.247	< 0.001
NO	75 (40.98%)	28 (15.30%)		
I/II	76 (41.53%)	81 (44.26%)		
III/IV	32 (17.49%)	74 (40.44%)		

*Expressed as (Median, Q1-Q3).

*CAS* Calcified aortic stenosis, *SBP* systolic blood pressure, *DBP* diastolic blood pressure, *HbA1c* glycosylated hemoglobin A1c, *NT-proBNP* N-terminal pro-brain natriuretic peptide, *Hs-cTn* high-sensitivity cardiac troponin, *CRP* C-reactive protein, *WBC* white blood count, *SUA* Serum Uric Acid, *FPG* fasting plasma glucose, *TC* total cholesterol, *TG* triglyceride. *AV* aortic valve, *TygI* triglyceride–glucose index, *HDL-C* high-density lipoprotein cholesterol, *LDL-C* low-density lipoprotein cholesterol, *LVEF* left ventricular ejection fraction, *T2DM* Type 2 Diabetes Mellitus.

Renal insufficiency, chronic kidney disease stages 1–3, *NYHA* New York Heart Association.

### Correlation analysis between the TyG index and CAS.

Table 2 describes the results of the logistic regression analysis. Univariate logistic regression analysis showed that the TyG index and CAS were statistically significant (OR, 0.553; 95% CI, 0.384–0.796; P = 0.001). After adjusting for age and sex in Model 1, the TyG index as a continuous variable was negatively correlated with CAS (OR, 0.537; 95% CI, 0.369–0.782; P = 0.001). In Model 2, after adjusting for age, sex, smoking, hypertension, type-2 diabetes mellitus, hyperlipidemia, HDL-C, LDL-C, and serum uric acid, we found that the TyG index, as a continuous variable, was still negatively correlated with CAS (OR, 0.434; 95% CI, 0.278–0.678; P < 0.001). We further used a trend test to analyze the stability of the trend. We divided the enrolled participants equally into three groups according to the tertiles of the TyG index as follows: the median of each group in the three groups was included in the equation as a continuous variable and further analyzed by trend test. As shown in Table 2, in the crude model, Model 1 and Model 2, with the increase in TyG index stage, the probability of CAS decreased, and there were significant differences (P < 0.05). The trend test further verified this view, in which the TyG index was negatively correlated with CAS.

**Table 2:** Relationship between TyG index and AS in different models.

Variable	Crude Model		Model I		Model II	
	OR (95%CI)	P-value	OR (95%CI)	P-value	OR (95%CI)	P-value
TyG index	0.553 (0.384, 0.796)	0.001	0.537 (0.369, 0.782)	0.001	0.434 (0.278, 0.678)	< 0.001
TyG index group						
6.63–8.14	Reference		Reference		Reference	
8.15–8.62	0.704 (0.423, 1.171	0.176	0.678 (0.401, 1.137)	0.139	0.623 (0.365 1.084)	0.095
8.66–11.00	0.496 (0.297, 0.827)	0.007	0.468 (0.275, 0.798)	0.005	0.368 (0.200, 0.678)	0.001
P for trend	0.008		0.005		0.001	

*CI* confidence interval.

Non-adjusted model adjust for: None.

Adjust I model adjust for: age, gender.

Adjust II model adjust for: age, gender, smoking, hypertension, T2DM, hyperlipidemia, HDL-C, LDL-C, and serum uric acid.

### Curve Fitting

**Figure 2:**
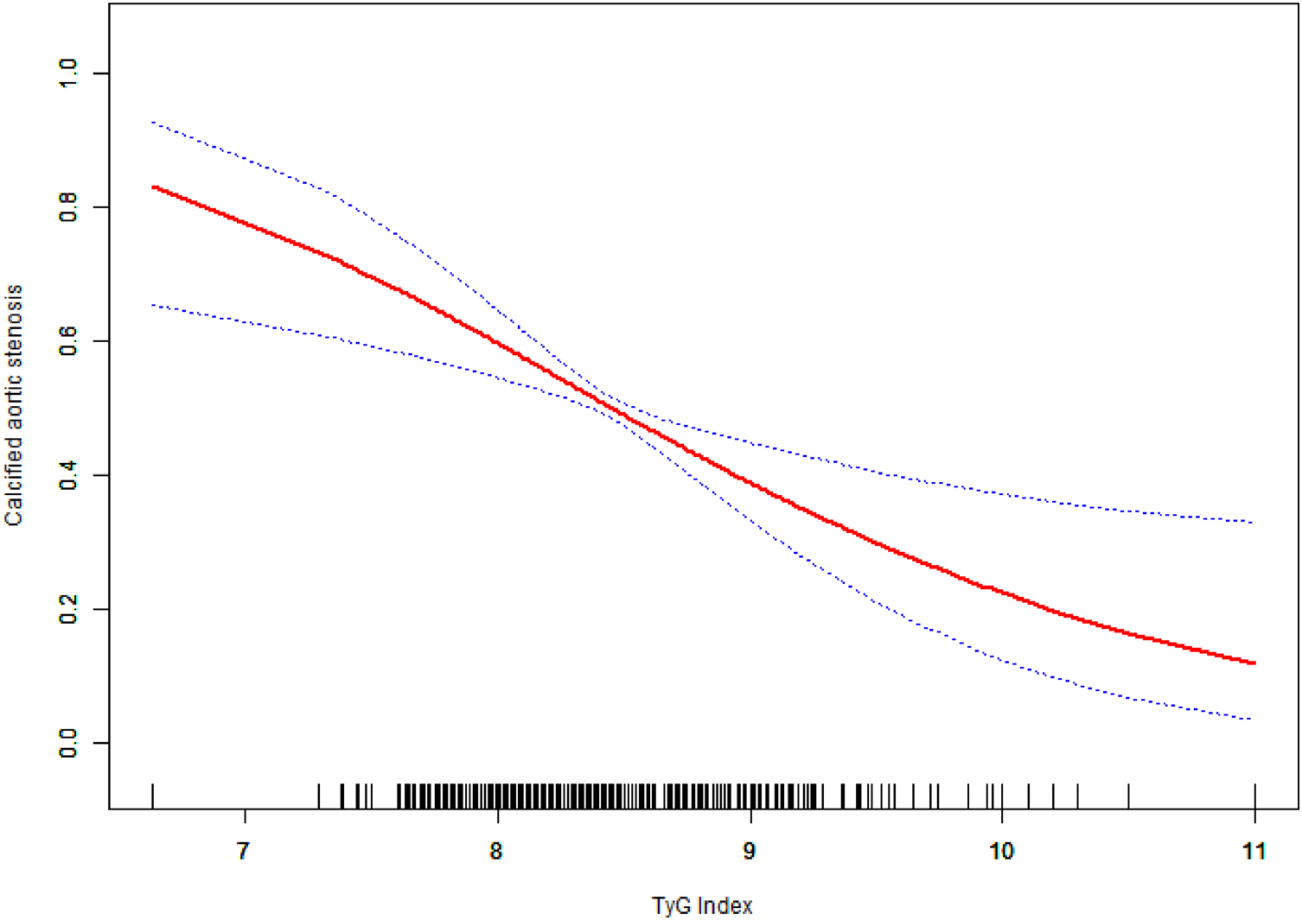
The relationship between TyG index and the CAS.

In the analysis of the correction between the TyG index and CAS, the GAM test results showed an approximately linear relationship between the TyG index and CAS after adjusting for age, sex, smoking, hypertension, type-2 diabetes mellitus, hyperlipidemia, HDL-C, LDL-C, and serum uric acid (p < 0.001). With the increase in the TyG index, the probability of CAS was reduced (Fig. 2). The solid red line represents the smooth curve fit between variables. Blue bands represent the 95% confidence interval from the fit.

### The correlation between different degrees of CAS and the TyG index

This study also analyzed the correlation between different degrees of CAS and the TyG index. As shown in Table 3, we could see that in unadjusted, Model 1 and Model 2, different degrees of aortic stenosis were negatively correlated with the TyG index. After adjusting for age, sex, smoking, hypertension, type-2 diabetes mellitus, hyperlipidemia, HDL-C, LDL-C, and serum uric acid, compared with those without aortic stenosis, the TyG index was negatively correlated with mild and severe calcific aortic stenosis, and the TyG index decreased by 0.201 units (P = 0.014) in the mild calcific aortic stenosis group, and decreased by 0.252 units (P < 0.001) in the severe calcific aortic stenosis group.

**Table 3:** The correlation between different degrees of CAS and TyG index.

Variable	Crude Model		Model I		Model II	
	β (95%CI)	P-value	β (95%CI)	P-value	β (95%CI)	P-value
CAS						
NO	Reference		Reference		Reference	
Mild	− 0.198 (− 0.382, − 0.014)	0.035	− 0.208 (− 0.391, − 0.024)	0.027	− 0.201 (− 0.362, − 0.041)	0.014
Moderate	− 0.128 (− 0.320, 0.063)	0.190	− 0.137 (− 0.325, 0.052)	0.155	− 0.136 (− 0.300, 0.029)	0.107
Severe	− 0.256 (− 0.411, − 0.100)	0.001	− 0.245 (− 0.399, − 0.092)	0.002	− 0.252 (− 0.391, − 0.113)	< 0.001

*CI* confidence interval.

Non-adjusted model adjust for: None.

Adjust I model adjust for: age, gender.

Adjust II model adjust for: age, gender, smoking, hypertension, T2DM, hyperlipidemia, HDL-C, LDL-C, and serum uric acid.

## Discussion

In this study, the TyG index was negatively correlated with CAS. After adjusting for age, sex, smoking, hypertension, T2DM, hyperlipidemia, HDL-C, LDL-C, and serum uric acid, the TyG index was still negatively correlated with CAS, which was further verified by a trend test. The TyG index is a reliable indicator of IR. This study was the first to observe the correlation between IR and CAS.

The aortic valve is composed of three layers: the ventricular layer on the ventricular side, the valvular layer on the aortic side and the cavernous layer that lubricates the other layers. These three layers are filled with valvular interstitial cells (VICs), and the entire lamellar structure is covered by endothelial cells^8^. The initial stage of CAS is similar to that of atherosclerosis, in which valve endothelial dysfunction is mainly caused by mechanical stress, inflammation, lipid deposition and so on. Under the stimulation of further oxidative stress, valvular interstitial cells (VICs) differentiate into myofibroblasts and osteoblast phenotypes, promoting calcification and osteogenic changes in the valve and further narrowing of the valve. However, previous studies have shown that the TyG index is positively correlated with coronary artery stenosis and peripheral artery disease^9,10^. Therefore, we believe that IR not only affects the first stage of the development of CAS but also participates in the progression of CAS.

Relevant studies have shown that the differentiation of aortic valvular interstitial cells (VICs) into osteoblasts is affected by a variety of factors. For example, DPP-4 inhibits the signaling pathway of IGF-1-IGFR and induces osteogenic differentiation of VIC by changing the structure of IGF-1; mechanical stress and inflammatory stimulation can promote the expression of osteogenic genes and calcification in normal and calcified VICs^11,14^. In addition, osteoblasts are also one of the target cells of insulin. Thomas L. Clemens et al. found that osteoblast differentiation can be regulated by inhibiting the known Runx2 inhibitor Twist2 in the insulin signaling pathway; meanwhile, the proliferation, survival and differentiation of osteoblasts are also regulated by the insulin-receptor signaling pathway^15^. A lack of insulin receptors increases the incidence of obesity and IR. IR is a disordered biological response to insulin stimulation through the destruction of different molecular pathways in target tissues^16^, which increases the level of oxidative stress by affecting the body's glucose metabolism and lipid metabolism. Therefore, we speculated that when the body has IR, the number of insulin receptors is reduced or less sensitive, and the insulin signaling pathway is damaged, which affects the survival and proliferation of osteoblasts.

In a study on the relationship between IR defined by the TyG index and BMD of different bones, it was found that in nondiabetic men aged ≥ 50 years old and postmenopausal women aged ≥ 50 years old, the TyG index was inversely correlated with BMD of the femoral neck^17^. The Fuglsang-Nielsen study found that P1NP, a marker of bone formation, was negatively correlated with IR, suggesting that there may be a relationship between the development of IR and low bone turnover^18^. ArunS Karlamangla's study on the longitudinal correlation between IR and bone mineral density changes showed that when IR increases over time, IR may be harmful to BMD (i.e., accelerate BMD loss)^19^. A number of studies have also proven that IR is negatively correlated with bone mass, femoral neck strength and lumbar bone density^20–22^ and is associated with an increased risk of osteoporosis^23,24^. The association between IR and BMD has also been observed in the diabetic population. Compared with diabetes patients with low IR, patients with type-2 diabetes with high IR have a lower BMD^25^.

In summary, we found that IR is negatively associated with CAS in elderly patients, which may be related to the impaired insulin-receptor signaling pathway associated with IR. Mechanical stimulation and oxidative stress can promote the expression of osteoblast genes in valvular stromal cells and promote the transformation of valvular cells into osteoblasts. The survival and proliferation of osteoblasts are regulated by the insulin-receptor signaling pathway. Therefore, we speculated that when the body has IR, the insulin-receptor signaling pathway is damaged, and the proliferation and survival of osteoblasts are inhibited, thus affecting the occurrence and development of calcifying aortic stenosis. Our study is the first to discover the correlation between IR and CAS in the elderly population and provides a new perspective for the occurrence and development of CAS. We also believe that more prospective studies are needed to verify this.

## Conclusion

Our study showed that the TyG index was negatively associated with CAS in elderly patients, which may be related to the impairment of insulin receptors and signaling pathways in IR.

### Strengths and limitations

To our knowledge, this is the first study to examine the association between TyG index and CAS in elderly patients. However, there are some limitations to the study. First, our study originates from single-center data and includes a relatively limited number of patients, which requires more relevant studies to participate. Second, because this is a cross-sectional study, we cannot infer causality in this study. Third, the subjects of our study included part of patients with type 2 diabetes and hyperlipidemia. In the regression analysis, we also adjusted these two variables. Therefore, future prospective studies are warranted to verify these findings.

## Data Availability

The datasets generated and analyzed during the current study are not publicly available due privacy and ethical restrictions but are available from the corresponding author on reasonable request.

## Abbreviations

CAS: Calcified aortic stenosis
SBP: Systolic blood pressure
DBP: Diastolic blood pressure
glycosylated hemoglobin A1c: HbA1c
NT-proBNP: N-terminal pro-brain natriuretic peptide
Hs-cTn: High-sensitivity cardiac troponin
CRP: C-reactive protein
WBC: White blood count
SUA: Serum Uric Acid
FPG: Fasting plasma glucose
TC: Total cholesterol
TG: Triglyceride
AV: Aortic valve
TygI: Triglyceride–glucose index
HDL-C: High-density lipoprotein cholesterol
LDL-C: Low-density lipoprotein cholesterol
LVEF: Left ventricular ejection fraction
T2DM: Type 2 Diabetes Mellitus, Renal insufficiency, chronic kidney disease stages 1–3
NYHA: New York Heart Association
